# Traditional Salads and Soups with Wild Plants as a Source of Antioxidants: A Comparative Chemical Analysis of Five Species Growing in Central Italy

**DOI:** 10.1155/2019/6782472

**Published:** 2019-03-05

**Authors:** Valentina Savo, Francois Salomone, Elena Mattoni, Daniela Tofani, Giulia Caneva

**Affiliations:** Department of Science, Roma Tre University, Viale G. Marconi 446, Rome, Italy

## Abstract

The interest and demand for nutraceuticals are rapidly increasing in many industrialized countries due to the emergence of health risks associated with the increased consumption of processed foods. Several wild Mediterranean plants used as traditional foods are an extraordinary source of nutraceutical substances with antioxidant properties. This study has two main aims: (1) to quantify the antioxidant properties of traditional wild food plants and (2) to determine if their use in soups (i.e., the cooking process) can alter their beneficial properties. We have evaluated the antioxidant capacity (ABTS, DPPH) and the Total Phenolic Content (Folin-Ciocalteu) of five herbaceous plants traditionally consumed in several areas of Central Italy: (*A*)* Reichardia picroides* (L.) Roth, (*B*)* Hypochaeris radicata *L., (*C*)* Cichorium intybus *L., (*D*)* Tordylium apulum* L., and (*E*)* Helminthotheca echioides *(L.) Holub. Our analyses show good levels of antioxidant capacity for all plants, with* Reichardia picroides* and* Helminthotheca echioides* having the highest levels. There is a high correlation between the antioxidant activity and the Total Phenolic Content especially in* Reichardia picroides* (R^2^=0.92) and* Hypochaeris radicata *(R^2^=0.93). Boiling the species caused a general decrease in the antioxidant activity and polyphenols. Our study confirms the health benefits of consuming wild plants, especially raw ones in salads. It also supports the use of ethnobotanical information to study and then promote the consumption of wild food plants.

## 1. Introduction

Wild food plants used in the traditional Mediterranean diet have received much attention in recent years for their nutraceutical properties and in particular for their content of antioxidant compounds [1–8]. Indeed, many studies have highlighted that a dietary antioxidant intake has a protective effect against free radical-related pathologies, such as cardiovascular diseases [9], diabetes [10], cancer [11, 12], and neurodegenerative diseases [13]. The morbidity of these diseases has increased in the last few decades in many industrialized countries [14] and has been often related, among other things, to shifts from traditional to western diets [15].

In the Mediterranean basin, ethnobotanical research has identified about 2,300 different wild plants and fungi taxa, which are still gathered and consumed as food [16]. Although in decline, the consumption of wild edible plants is still common in various areas in Italy, where they are consumed because they are considered healthy and tasty [17–19], and also because they are linked to tradition and culture [18, 20]. The traditional uses of wild food plants may contribute to the health benefits associated with the Mediterranean diet and, as a consequence, studies on their phytochemistry can validate their nutraceutical properties [5–7]. This is also supported by the fact that recent studies have highlighted that the protective effect of nutraceuticals against various diseases is linked to the association of several phytochemical molecules at low concentrations, as it occurs naturally in the diet, rather than to the ingestion of individual molecules at high concentrations, as occurs in pills of dietary supplements [21–23]. Research also supports the importance of investigating the antioxidant properties of the plant part that is actually consumed, rather than focusing the attention on the effects of individual compounds [1]. Finally, although several compounds may contribute to the antioxidant properties in complex systems [24, 25], polyphenols are often considered the primary source of the antioxidant activity [26–30] but few data support a precise correlation [29, 31, 32].

Consequently, this study is aimed at (i) evaluating and comparing the total antioxidant capacity and the Total Phenolic Content in different species of wild plants, traditionally consumed either raw or cooked in Central Italy; (ii) evaluating the relationship between the antioxidant capacity and the phenolic compounds contained in plant extracts to verify whether or not the phenolic constituents are responsible for the antioxidant activity of the species.

## 2. Materials and Methods

### 2.1. Sampling and Plants Collection

We sampled five wild plants: (*A*)* Reichardia picroides* (L.) Roth (Asteraceae), (*B*)* Hypochaeris radicata *L. (Asteraceae), (*C*)* Cichorium intybus *L. (Asteraceae), (*D*)* Tordylium apulum* L. (Apiaceae), and (*E*)* Helminthotheca echioides *(L.) Holub. (Asteraceae). We selected these species because they are, to different extents, purposively consumed among local communities in Italy because they are considered to have positive effects on the health [19, 33–35].

We gathered four specimens of each species (20 samples) in the Tolfa Mountain area (70 Km north-west of Rome). We selected only specimens with vigorous growth, collected in areas with similar soil characteristics, within an altitudinal range of 350-400 m a.s.l, growing in flat areas to eliminate the influence of different exposures to the sun. The samples were carefully extracted, and they were carried intactly, along with their soil, to the laboratory of Roma Tre University, to keep the leaves alive until they were cut.

### 2.2. Chemical Analysis

*Reagents.* All chemicals used were of analytical grade. The used solvents and reagents were purchased from Sigma Aldrich (Germany).

### 2.3. Preparation of Extracts from Crude Plants

For each sample collected, 1 g of leaves was cut from the plant, gently cleaned with some paper, and weighed. We then put the leaves in a Falcon tube and poured 10 mL of liquid nitrogen inside. The leaves immediately became hard and fragile and then they were crushed to dust. We put the Falcon tube in a lyophilizer (Christ Alpha 1-2 b. Braun biotech international. Savant refrigerated condensation trap RT 100) until the weight of the plant material was constant. We then added 5 ml of methanol to the Falcon tube. We mixed the solution using an ultrasound apparatus (Sonica Soltec 2002MH) for 60 minutes at 30°C. Afterwards, the solution was filtered in an Eppendorf test tube (final volume 10 ml), put in a nitrogen atmosphere, and left in the freezer (-20°C) until analysis. We performed three analyses for each plant species (namely, DPPH analysis, ABTS analysis, and Total Phenolic Content).

### 2.4. Preparation of Extracts from Cooked Plants

We collected 1 g of leaves from each plant, put the leaves in a small beaker containing 10 ml of boiling water, and left them to cook for 5 minutes. After that, the leaves were recovered and gently dried over a clean piece of paper. We then put the leaves in a Falcon tube and poured 10 mL of liquid nitrogen inside and continued the procedure as previously described for the crude material (i.e., leaves were frozen with liquid nitrogen, crushed to dust, lyophilized to remove the water, and extracted with 5 ml of methanol in an ultrasound apparatus for 60 minutes at 30°C; the solution was filtered and put in nitrogen atmosphere and then placed in the freezer). We performed three analyses for each plant species (namely, DPPH analysis, ABTS analysis, and Total Phenolic Content).

### 2.5. DPPH Analysis

We performed a DPPH analysis of the samples, with some adjustments, following the method described by Brand-Williams et al. [36]. We prepared a 75 *μ*M solution of DPPH in methanol. Plant extracts were diluted and analyzed at three different final concentrations ranging from 1.5 to 5 mg/ml. We added 50 *μ*l of each sample solution to 0.950 ml of the DPPH solution and left them in the dark. After 30 minutes, we measured the absorbance of the samples at 517 nm using the Shimadzu UV-2401 PC spectrophotometer. We used 50 *μ*L of pure ethanol as a control. We repeated four measurements for each plant sample.

Subsequently, we plotted the percentages of DPPH inhibition* vs* the antioxidant concentrations and elaborated linear regressions using the Graphpad Prism 4.1 program (http://www.graphad.com). From each graph, we extrapolated IC_50_ values as the concentration of the sample that halves the DPPH radical absorbance. Plants with a lower IC_50_ value contained higher levels of antioxidants. We performed statistical analyses applying Student's t-test and ANOVA as analyses of variance for the IC_50_ values. We also calculated the Antiradical Activity (ARA), as the inverse of IC_50_. We calculated all values, including relative errors, through the propagation of uncertainty.

### 2.6. ABTS Analysis

To measure the antioxidant capacity of all samples, we followed the method of Pellegrini et al. [37], with some adjustments. We prepared the ABTS radical cation solution mixing 10 ml of 7.0 mM aqueous solution of ABTS with 10 ml of 2.28 mM aqueous solution of K_2_S_2_O_8_ and diluting it to a 25 ml final volume (ABTS^+•^ solution). Then, we left the solution at room temperature overnight. Before the analysis, we diluted the ABTS^+•^ solution with ethanol to reach an absorbance of 0.70±0.20. In each analysis, we added 10 *μ*l of plant extracts to 1 ml of the diluted ABTS^+•^ solution. All plant extracts were analyzed in ethanol (0.2% of water) at room temperature using three different final concentrations ranging from 0.1 to 1.0 mg/ml. We measured the extent of colour fading after 3 minutes at *λ*=734 nm using a Shimadzu UV-2401 PC spectrophotometer. We performed four measurements for each concentration. We also run solvent blanks, and we used Trolox (6-Hydroxy-2,5,7,8-tetramethylchroman-2-carboxylic acid) as a reference antioxidant (Trolox is the hydrophilic derivative of alpha-tocopherol).

We calculated the dose-response curves as the percentage of absorbance decrease (% ABTS inhibition) against the amount of antioxidant concentration for each plant collected. We performed linear regressions and extrapolated slopes of the dose-response relationship using the Graphpad Prism 4.1 program (http://www.graphad.com). The level of significance for linear regressions was* p* <0.005 for all datasets for either the crude or the cooked samples. We reported the antioxidant capacities as Trolox Equivalent Antioxidant Capacity (TEAC), defined as the concentration (mmol/l) of Trolox having the equivalent antioxidant capacity of 1 kgfw/l solution of the plant extract under investigation. We calculated the average TEAC values for each group of plants, and we performed statistical analyses applying Student's t-test and ANOVA as analyses of variance of the TEAC values.

### 2.7. Total Phenolic Content (TPC)

The Folin-Ciocalteu reagent assay [38] was used to determine the Total Phenolic Content (TPC). As a first step, we diluted 2.5 ml of Folin-Ciocalteu commercial reagent to 25 ml with deionized water obtaining a new solution (solution A). We mixed 0.10 ml of each plant sample with 0.75 ml of solution A and let to rest for three minutes at 25°C before adding 0.75 ml of a saturated sodium carbonate solution. We let the new mixed solution rest for another 120 minutes before measuring the absorbance at 725 nm. Analyses were performed in quadruplicate for each plant collected. We used Gallic acid as a standard for the calibration curve. The Total Phenolic Content (TPC) was expressed as Gallic Acid Equivalents (GAE) i.e., the mg of Gallic acid corresponding to the polyphenols present in l g of dry plant material. As for the other analyses, we calculated the average TPC value for each plant and performed Student's t-test and analysis of variance (ANOVA).

### 2.8. Correlation between Antioxidant Capacity and Total Phenolic Content

We correlated the antioxidant capacity and Total Phenolic Content. Specifically, we used TPC and Antiradical Activity (ARA) to calculate the antioxidant capacity as a function of the presence of phenolic compounds in the plants. We analyzed the data as a whole and then as disaggregated sets for the four plant samples of each species. We elaborated linear regressions and determined slopes using the Graphpad Prism 4.1 program (http://www.graphad.com).

### 2.9. Statistical Analyses and Literature Search

All data were expressed as mean ± standard error (SE). To test differences among DPPH, ABTS, and TBC, we performed a series of one-way analyses of variance (ANOVA) and Bonferroni's Multiple Comparison Test. Statistical analyses were performed using the Graphpad Prism 4.1 program (http://www.graphad.com).

We carried out a literature search on the antioxidant capacity and phenolic content of the selected plants. We used common scientific literature search engines and databases (i.e., Google Scholar, Pubmed, and Science Direct) using as keywords the scientific names of the plants and the names of the various analytical tests. Subsequently, when possible, we compared the antioxidant activity and polyphenol contents with literature data and possible reasons behind significant differences in values were discussed.

## 3. Results and Discussion

### 3.1. Ethnobotanical Sampling and Plants Collection

In Italy, the gathering and use of wild edible species, even if it is decreasing and practiced mainly by older people, is still widespread throughout the entire country, mostly in rural areas. The plants we selected are commonly consumed raw or cooked in Central Italy to prepare various traditional dishes [17, 18, 39]. The Tolfa area, where we gathered the samples, is considered very important from an ethnobotanical point of view [40, 41]. In the area, the leaves of these plants are commonly consumed fresh in salads or cooked in a tasty soup called “*Acquacotta*”. For this reason, we sampled and analyzed four plants from each of the five wild species* Reichardia picroides* (L.) Roth (Asteraceae) (*A*),* Hypochaeris radicata* L. (Asteraceae) (*B*),* Cichorium intybus* L. (Asteraceae) (*C*),* Tordylium apulum* L. (Apiaceae) (*D*), and* Helminthotheca echioides* (L.) Holub. (Asteraceae) (*E*). Furthermore, three plants* Hypochaeris radicata *(*BC*),* Cichorium intybus* (*CC*), and* Helminthotheca echioides *(*EC*) were also analyzed after cooking. In Italy, all five plants are traditionally used, besides as food, also for their medicinal properties; i.e., they are used to treat heart problems, infections, and diabetes and as a depurative [7, 17, 42]. These plants have a wide distribution in Italy and can grow in many different habitats, mostly arid and ruderal [43]. As such, their cultivation could be promoted in marginal and arid lands or abandoned fields [44, 45].

### 3.2. DPPH Assay

**Figure 1:**
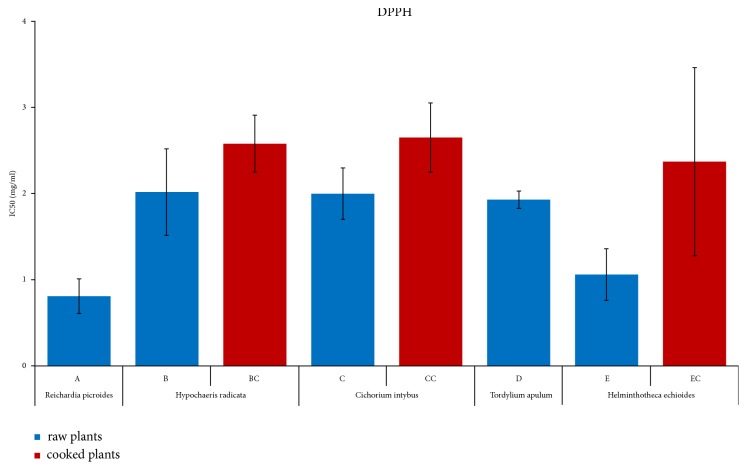
*DPPH assay of the raw and cooked plant extracts.* The average antioxidant capacity for four plants of each species either raw (*A-E*) or cooked (*BC*,* CC*, and* EC*) is presented as IC_50,_ i.e., the inhibition concentration that halves the DPPH radical activity. Lower IC_50_ values indicate higher antioxidant capacity. Statistical analyses of all average data were performed using ANOVA.

The DPPH radical scavenging assay is one of the most extensively used methods for estimating the antioxidant efficacy of molecules and plant samples [46]. We reported graphically the extent of the antioxidant capacity that was determined as the amount of antioxidant that halves the DPPH radical concentration (IC_50_) of the four samples of each species in Figures S1 and S2 of Supporting Information (S.I.). In Figure 1 and Tables 1 and 2, we showed the obtained IC_50_ average values for the five fresh plants (*A-E*) and the three cooked plants (*BC*,* CC*, and* EC*). All the analyzed plants showed antioxidant properties. In Table 1, it is possible to observe that, in the fresh samples, IC_50_ ranges from 2.69±0.05 mg/ml for* Hypochoeris radicata* (*B1*) to the higher 0.57±0.02 mg/ml for the antioxidant capacity of* Reichardia picroides* (*A3*). The cooking caused a general lowering of the antioxidant activity in all samples. In this case, IC_50_ ranged from 3.1±0.3 mg/ml of* Cichorium intybus* (*CC3*) to 1.73±0.07 mg/ml of* Helminthotheca echioides *(*EC4*) (Table 2). The comparison of fresh* Cichorium intybus *IC_50_ values with those reported in literature was, in some cases, difficult as published data dealt with antioxidant analyses of different parts of the plant (0.2-1 mg/ml for roots [47]) or cultivated varieties (5.7 nmol Trolox/mg of fresh weight for red chicory [48]). Some works, instead, reported lower values of IC_50_ for wild plants of* Cichorium intybus *(1.11 mg/ml [6]). The literature on the antioxidant capacity of cooked plants was instead quite scarce (i.e., [49]).

Statistical analyses, performed applying Student's t-test to the samples, gave a level of significance of* p* < 0.005 for all the fresh samples and* p* < 0.05 for the cooked samples (blue bars in Figures S1 and S2). The average IC_50_ value for each group of crude samples showed a level of significance of* p* < 0.001 and of* p* < 0.05 for the cooked samples (Figure 1). The Statistical Analysis of Variation (ANOVA) of IC_50_ values showed a significant difference between the fresh plant species: the IC_50_ value of* A* and* E* proved to be similar to each other but significantly different from* B*,* C,* and* D*. Instead, the* B*,* C,* and* D* species showed IC_50_ values that were not statistically different from each other (Figure S1). For the cooked plants, the differences among the IC_50_ values of all the samples were not statistically significant (Figure S2).

### 3.3. ABTS Assay

**Figure 2:**
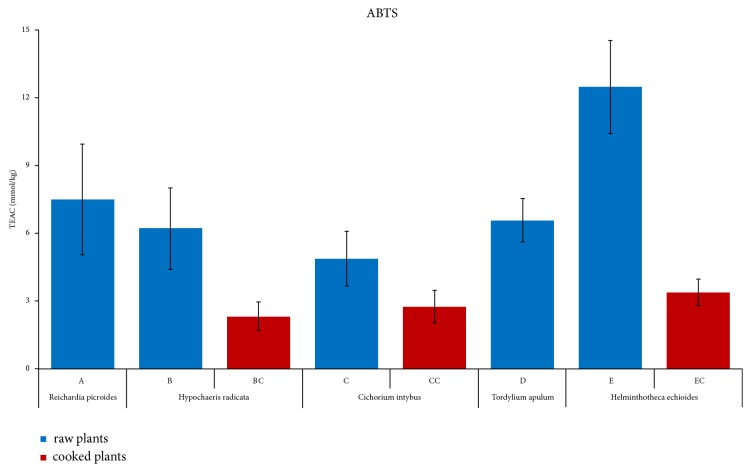
*ABTS assay of the raw and cooked plant extracts.* The average antioxidant capacity for four plants of each species either raw (*A-E*) or cooked (*BC*,* CC*, and* EC*) is presented as TEAC, i.e., mmol of Trolox equivalent* per* kg of fresh weight (kgfw). Statistical analyses of all average data were performed using ANOVA.

The ABTS assay also supported the antioxidant properties of the selected plants. This method of analysis is generally more sensitive than the DPPH assay to phenolic and flavonoid contents [50]. In Figure 2, we showed the relative average values expressed as Trolox Equivalent Antioxidant Capacity (TEAC) for each species either crude or cooked and their relative errors calculated with the propagation of uncertainty (see also Tables 1 and 2). The TEAC values of crude samples ranged from the lower 3.7±0.1 mmol/kgfw of* Cichorium intybus* (*C4*) to 15.4±0.5 mmol/kgfw of* Helminthotheca echioides* (*E1*) (Table 1), while for the cooked leaves, TEAC values spanned from 1.79±0.06 mmol/kgfw of* Hypochaeris radicata *(*BC1*) to 4.16±0.1 mmol/kgfw of* Helminthotheca echioides *(*EC4*) (Table 2). As in the DPPH analysis, the lower TEAC values of the cooked samples could be explained by the instability of phenolic antioxidants at high temperatures. The average TEAC value of 4.9±1 mmol/kgfw obtained from the analysis of the four samples of the crude* Cichorium intybus* is coherent with literature data (see also [51] for the wild specimens). Since* Cichorium intybus* is generally considered as having a significant level of antioxidant capacity [51], the higher TEAC values obtained for all the other examined species (crude samples) supported their good antioxidant capacity. Nevertheless, the average TEAC value for each species showed a high standard deviation (Figures S3 and S4). Other statistical analyses, instead, confirmed the robustness of the data and attested the antioxidant capacity of the plants. The level of significance for the Student's t-test was* p* < 0.01 for the crude samples and* p* < 0.05 for the cooked samples. The Analysis of Variation (ANOVA) revealed that significant differences in TEAC values only exist between* E* and other crude plant samples. In fact,* Helminthotheca echioides *(*E*) showed an average TEAC value of 12±2 mmol/kgfw, more than 2.5 times higher than that of* Cichorium intybus *(Figure 2), supporting its good antioxidant capacity. All the other TEAC values of crude (*A*,* B*,* C*, and* D*) or cooked (*BC*,* CC*, and* EC*) samples were not statistically different from each other.

### 3.4. Total Phenolic Content

**Figure 3:**
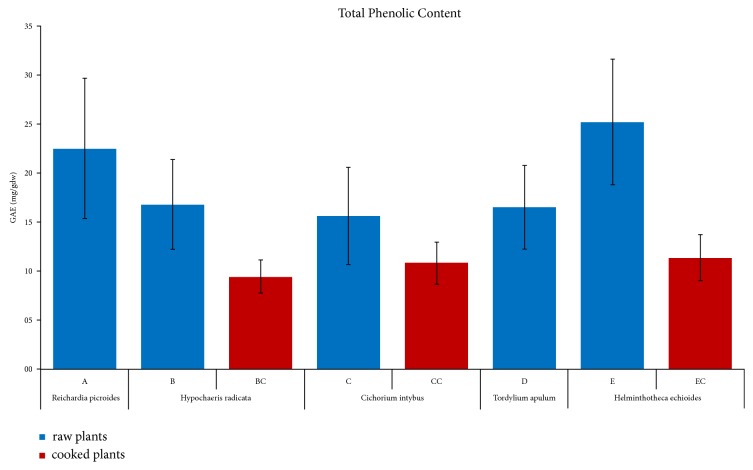
*Total Phenolic Content of the raw and cooked plant extracts determined using the Folin-Ciocalteu assay.* The averages data of all raw (*A-E*) and cooked (*BC*,* CC*, and* EC*) species are presented as Gallic Acid Equivalents (GAE); i.e., mg of Gallic acid corresponding to the polyphenols contained* per* gram of dry weight (gdw). Statistical analyses of all average data were performed using ANOVA.

The average Total Phenolic Content (TPC) of the plant extracts is presented in Figure 3, while the TPC values for all the samples are provided in the Figures S5 and S6. The crude plant extracts showed a polyphenol level ranging from 10.5±0.4 mg GAE/gdw in* Cichorium intybus* (*C4*) to 33±1 mg GAE/gdw in* Helminthotheca echioides* (*E1*) (Table 1). On the other hand, cooked leaves displayed a lower TPC ranging from 8.0±0.3 mg GAE/gdw (*EC3*) to 13.2±0.7 (*EC2*) mg GAE/gdw, both measured in samples of* Helminthotheca echioides*. Literature data about crude extracts of* Cichorium intybus* indicated a lower phenolic content (0.66 mg GAE/gdw [6] or higher (22.6±1.0 mg GAE/gdw [52]) than the mean value of the four crude plant extracts analyzed (16±5 mg GAE/gdw). This could be due either to the intrinsic differences in the growing environment of the plant (i.e., soil, exposure to solar radiation, and hydric supply [52]) or to different sample preparations that could have safeguarded polyphenols from degradation.

TPC values and their relative errors, calculated through the propagation of uncertainty, are reported in Tables 1 and 2. The level of significance is* p* < 0.05 for all data. The ANOVA test for the average TPC values confirmed a low variation in phenolic concentrations among the various plants either crude or cooked: these concentrations were not statistically different from each other except to some marginal differences between* E* and* B* or* C* and* D* (crude samples).

### 3.5. Correlation between Antioxidant Capacity and Total Phenolic Content

**Figure 4:**
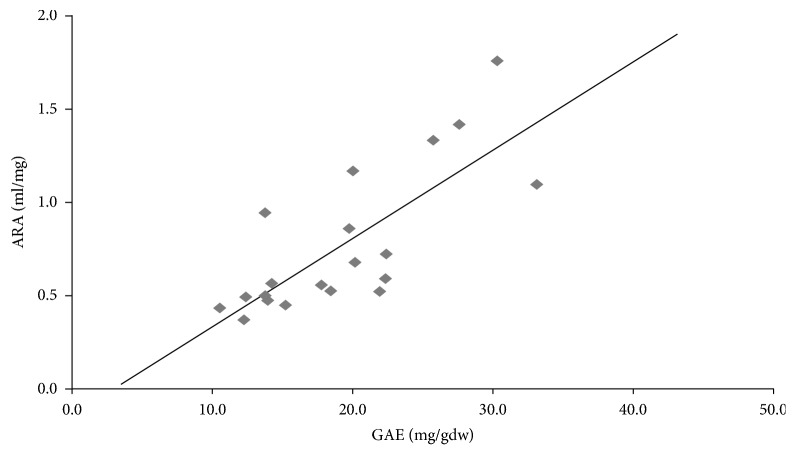
*Correlation between the Antiradical Activity (ARA) and Total Phenolic Content (TPC) for all data of the crude plant extracts:* y = 0.047x - 0.14; R^2^ = 0.59; p <0.0001. Linear regressions and best fit values were calculated using the Graphpad Prism 4.1 program (http://www.graphad.com).

**Figure 5:**
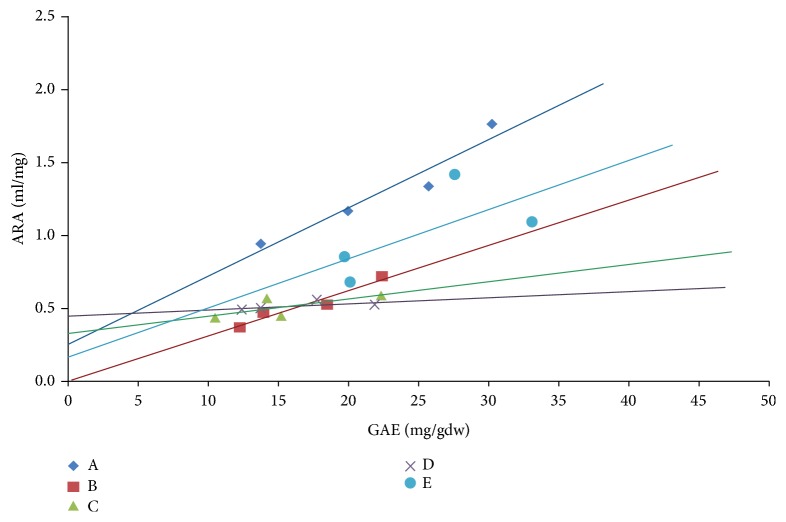
*Correlation between the Antiradical Activity (ARA) and Total Phenolic Content for each raw species (A-E). A Reichardia picroides* (◆) y = 0.047x +0.25; R^2^ = 0.92;* B Hypochaeris radicata *(■) y = 0.031x +0.009; R^2^ = 0.93;* C Cichorium intybus *(Δ) y = 0.011x +0.34; R^2^ = 0.52;* D Tordylium apulum* (x) y = 0.004x +0.45; R^2^ = 0.33; and* E Helminthotheca echioides *(•) y = 0.03x+0.25; R^2^ = 0.45.* A* (p< 0.05),* B* (p< 0.05),* C* (p< 0.1),* D* (p< 0.3), and* E* (p< 0.1). Linear regressions and best fit values were calculated using the Grahpad Prism 4 program (http://www.graphad.com).

The Antiradical activity (ARA), as the inverse of IC_50_, is reported in Table 1 and was used to correlate TPC with the antioxidant capacity of crude samples (Figures 4 and 5). In Figure 4, we showed the graph plotting the values of ARA and TPC of all the crude plant samples under study. The graph showed a linear correlation (p<0.0001) with a slope of 0.047±0.009 and -0.1±0.2 as *y* intercept. As regards Figure 4, the value of R^2^ (0.59) suggests a good linear correlation between the two variables (see [6]). In Figure 5, we showed the same linear correlations between ARA and TPC but with data disaggregated for the four plant samples of each species. For* Reichardia picroides A* (R^2^=0.92) and* Hypochaeris radicata B* (R^2^=0.93) the antioxidant capacity proved to be linearly dependent on TPC while a weaker correlation was observed with* Cichorium intybus* (*C*),* Tordylium apulum* (*D*), and* Helminthotheca echioides *(*E*). The slope of each curve correlates the antioxidant capacity with the polyphenols contained in the plant: the higher the value of the slope the higher the antioxidant capacity of the polyphenols in the samples. Furthermore,* Hypochaeris radicata* (*B*) exhibited an antioxidant activity completely dependent on polyphenolic compounds, as shown by *y* intercepts tending towards zero in the absence of polyphenolic compounds (*x*=0), whereas the antioxidant capacity of* Reichardia picroides* (*A*),* Cichorium intybus *(*C*),* Tordylium apulum* (*D*), and* Helminthotheca echioides *(*E*) is probably due to other kinds of antioxidants (i.e., carotenoids), as shown by *y* intercepts different from zero at* x*=0.

Our results show that the examined wild food plants have a good antioxidant activity (see [53]), although with some differences among plants, and a certain variability among samples of the same species. Among the analyzed plants,* Helminthotheca echioides* (*E*) and* Reichardia picroides* (*A*) have shown the best performances.* Helminthotheca echioides* (*E*) exhibited the highest antioxidant capacity using either the ABTS or the DPPH assays, coupled with the highest polyphenolic content.

The antioxidant capacity and the phenolic content are correlated in the plants under study. A strong correlation exists in* Reichardia picroides A* (R^2^=0.92) and* Hypochaeris radicata B* (R^2^=0.93), instead, in* Cichorium intybus* (*C*),* Tordylium apulum* (*D*), and* Helminthotheca echioides* (*E*) the correlation decreases due to the likely presence of nonpolyphenolic antioxidants. Similarly, Dalar et al. [54] found a correlation between total phenolics and antioxidant capacity in their experiments, but they also detected a significant level of other redox-active compounds besides phenolics. In another study, instead, the contribution of phenolic components to the antioxidant capacity was found to be at only 58% [55].

## 4. Conclusions

Our study demonstrated that the examined wild food plants, consumed in traditional recipes in Central Italy, are indeed rich in antioxidant compounds. Ethnobotanical research might guide the study and then the revitalization of a healthy Mediterranean diet that incorporates wild plants with antioxidant capacity. We also verified that this antioxidant capacity is mainly correlated with the presence of phenolic compounds although further analysis (i.e., the identification of the individual constituents of the mixture by HPLC or LC-MS) would better determine the presence of other antioxidant compounds. We were also able to assess that the way food is consumed (raw or cooked) can significantly alter the health benefits of the ingested plants. In view of the growing demand for food rich in natural antioxidants by the global market, the use of ethnobotanical information to study and then promote new agronomic products from wild food plants could constitute an important way to produce income in many rural regions.

## Figures and Tables

**Table 1:** Summary table with all the results obtained from the DPPH, ABTS, and Folin-Ciocalteu assays on the extracts of the crude plants.

Plant	Sample	DPPH IC50	ARA	ABTS TEAC	FOLIN GAE
		(mg/ml)	(ml/mg)	(mmol/kgfw)	(mg /gdw)
*Reichardia picroides*	*A1*	0.86 ± 0.03	1.17 ± 0.04	11.2 ± 0.3	20.0 ± 0.7
	*A2*	1.06 ± 0.03	0.94 ± 0.03	6.2 ± 0.2	13.8 ± 0.5
	*A3*	0.57 ± 0.02	1.76 ± 0.05	6.4 ± 0.2	30 ± 1
	*A4*	0.75 ± 0.07	1.33 ± 0.12	6.2 ± 0.2	25.8 ± 0.9
*Hypochaeris radicata*	*B1*	2.69 ± 0.05	0.37 ± 0.01	4.5 ± 0.1	12.2 ± 0.4
	*B2*	1.90 ± 0.04	0.53 ± 0.01	5.6 ± 0.2	18.4 ± 0.7
	*B3*	1.39 ± 0.07	0.72 ± 0.04	8.8 ± 0.3	22.4 ± 0.8
	*B4*	2.10 ± 0.07	0.48 ± 0.02	5.9 ± 0.2	14.0 ± 0.5
*Cichorium intybus*	*C1*	2.23 ± 0.02	0.45 ± 0.00	3.9 ± 0.1	15.2 ± 0.6
	*C2*	1.69 ± 0.08	0.59 ± 0.03	6.1 ± 0.2	22.4 ± 0.8
	*C3*	1.76 ± 0.03	0.57 ± 0.01	5.8 ± 0.2	14.2 ± 0.5
	*C4*	2.30 ± 0.03	0.44 ± 0.01	3.7 ± 0.1	10.5 ± 0.4
*Tordylium apulum*	*D1*	2.00 ± 0.05	0.50 ± 0.01	6.1 ± 0.2	13.8 ± 0.5
	*D2*	1.79 ± 0.01	0.56 ± 0.00	8.0 ± 0.3	17.8 ± 0.7
	*D3*	1.91 ± 0.12	0.52 ± 0.03	6.1 ± 0.2	21.9 ± 0.8
	*D4*	2.03 ± 0.06	0.49 ± 0.01	6.0 ± 0.2	12.4 ± 0.5
*Helminthotheca echioides*	*E1*	0.91 ± 0.01	1.09 ± 0.01	15.4 ± 0.5	33 ± 1
	*E2*	1.16 ± 0.04	0.86 ± 0.03	10.7 ± 0.3	19.8 ± 0.7
	*E3*	0.71 ± 0.02	1.42 ± 0.04	12.1 ± 0.4	27.6 ± 1.0
	*E4*	1.47 ± 0.07	0.68 ± 0.03	11.6 ± 0.4	20.2 ± 0.7

**Table 2:** Summary table with all the results obtained from the DPPH, ABTS, and Folin-Ciocalteu assays on the extracts of the cooked plants.

Plant	Sample	DPPH IC50	ARA	ABTS TEAC	FOLIN GAE
		(mg/ml)	(ml/mg)	(mmol/kgfw)	(mg /gdw)
*Hypochaeris radicata*	*BC1*	2.48 ± 0.05	0.40 ± 0.01	1.79 ± 0.06	8.1 ± 0.3
	*BC2*	2.2 ± 0.2	0.45 ± 0.04	2.20 ± 0.07	11.8 ± 0.4
	*BC3*	2.0 ± 0.3	0.50 ± 0.08	3.2 ± 0.1	8.5 ± 0.5
	*BC4*	2.64 ± 0.01	0.38 ± 0.00	2.07 ± 0.06	9.2 ± 0.3
*Cichorium intybus*	*CC1*	2.70 ± 0.04	0.37 ± 0.01	2.68 ± 0.08	12.9 ± 0.5
	*CC2*	2.37 ± 0.06	0.42 ± 0.01	2.53 ± 0.08	9.7 ± 0.4
	*CC3*	3.1 ± 0.3	0.32 ± 0.03	2.05 ± 0.06	8.4 ± 0.3
	*CC4*	2.3 ± 0.1	0.43 ± 0.02	3.8 ± 0.1	12.2 ± 0.4
*Helminthotheca echioides*	*EC1*	3.0 ± 0.4	0.33 ± 0.04	3.5 ± 0.1	11.4 ± 0.4
	*EC2*	1.8 ± 0.1	0.56 ± 0.04	2.91 ± 0.09	13.2 ± 0.7
	*EC3*	2.5 ± 0.1	0.40 ± 0.02	2.95 ± 0.09	8.0 ± 0.3
	*EC4*	1.73 ± 0.07	0.58 ± 0.02	4.2 ± 0.1	12.8 ± 0.5

## Data Availability

The data used to support the findings of this study are included within the article and the supplementary information files.

## Abbreviations

DPPH: 2,2-diphenyl-1-picrylhydrazyl radical
ANOVA: Analysis of variance
ARA: Antiradical activity
ABTS: 2,2′-azinobis(3-ethylbenzothiazoline-6-sulfonic acid) diammonium salt
TEAC: Trolox equivalent antioxidant capacity
Trolox: 6-Hydroxy-2,5,7,8-tetramethylchroman-2-carboxylic acid
TPC: Total phenolic content
GAE: Gallic acid equivalents
kgfw: Kilograms fresh weight
gdw: Grams dry weight
SE: Standard error

## Supplementary Materials

Supplementary Materials Figure S1.* DPPH assay of the crude plant extracts.* Data of each plant sample and averages of all species (*A-E*) are presented as IC_50_ i.e., the inhibition concentration that halves the DPPH radical activity. Lower IC_50_ values indicate higher antioxidant capacity. Statistical analyses of all average data were performed using ANOVA. Values for* A* and* E* resulted not statistically different from each other but different from all other values (p < 0.01) except* E* versus* B* (p<0.05). Figure S2.* DPPH assay of the cooked plant extracts*. Data of plant samples and averages of species (*BC*,* CC,* and* EC*) are presented as IC_50_ i.e., the inhibition concentration that halves the DPPH radical activity. Lower IC_50_ values indicate higher antioxidant capacity. Statistical analyses of all average data were performed using ANOVA. Values resulted not statistically different from each other. Figure S3.* ABTS assay of the crude plant extracts.* Data of each plant sample and averages of all species (*A-E*) are presented as TEAC i.e., mmoles of Trolox equivalent* per* kilogram of fresh weight (kgfw). Statistical analyses of all average data were performed using ANOVA. All average data resulted not statistically different from each other except for* E* that was statistically different from* A* (p< 0.05),* B* (p< 0.01),* C* (p< 0.001), and* D* (p< 0.01). Figure S4.* ABTS assay of the cooked plant extracts.* Data of plant samples and averages of species (*BC*,* CC* and* EC*) are presented as TEAC, i.e., mmoles of Trolox equivalent* per* kilogram of fresh weight (kgfw). Statistical analyses of all average data were performed using ANOVA. All average data resulted not statistically different from each other. Figure S5.* Total Phenolic Content of the crude plant extracts determined using the Folin-Ciocalteu assay.* Data of each plant sample and averages of all species (*A-E*) are presented as mg of Gallic acid* per* gram of dry weight (gdw). Statistical analyses of all average data were performed using ANOVA. All average data resulted not statistically different from each other except to some marginal differences between* E* and* B* or* C* and* D*. Figure S6.* Total Phenolic Content of the cooked plant extracts determined using the Folin-Ciocalteu assay.* Data of plant samples and averages of species (*BC*,* CC* and* EC*) are presented as mg of Gallic acid* per* gram of dry weight (gdw). Statistical analyses of all average data were performed using ANOVA. All average data resulted not statistically different from each other.
