# Supplementary material for: Traditional Salads and Soups with Wild Plants as a Source of Antioxidants: A Comparative Chemical Analysis of Five Species Growing in Central Italy
Source: Evid Based Complement Alternat Med. 2019 Mar 5;2019:6782472. doi: 10.1155/2019/6782472 (PMC6425424; doi:10.1155/2019/6782472)
Supplement: Supplementary Materials — Figure S1. DPPH assay of the crude plant extracts. Data of each plant sample and averages of all species (A-E) are presented as IC50 i.e., the inhibition concentration that halves the DPPH radical activity. Lower IC50 values indicate higher antioxidant capacity. Statistical analyses of all average data were performed using ANOVA. Values for A and E resulted not statistically different from each other but different from all other values (p < 0.01) except E versus B (p<0.05). Figure S2. DPPH assay of the cooked plant extracts. Data of plant samples and averages of species (BC, CC, and EC) are presented as IC50 i.e., the inhibition concentration that halves the DPPH radical activity. Lower IC50 values indicate higher antioxidant capacity. Statistical analyses of all average data were performed using ANOVA. Values resulted not statistically different from each other. Figure S3. ABTS assay of the crude plant extracts. Data of each plant sample and averages of all species (A-E) are presented as TEAC i.e., mmoles of Trolox equivalent per kilogram of fresh weight (kgfw). Statistical analyses of all average data were performed using ANOVA. All average data resulted not statistically different from each other except for E that was statistically different from A (p< 0.05), B (p< 0.01), C (p< 0.001), and D (p< 0.01). Figure S4. ABTS assay of the cooked plant extracts. Data of plant samples and averages of species (BC, CC and EC) are presented as TEAC, i.e., mmoles of Trolox equivalent per kilogram of fresh weight (kgfw). Statistical analyses of all average data were performed using ANOVA. All average data resulted not statistically different from each other. Figure S5. Total Phenolic Content of the crude plant extracts determined using the Folin-Ciocalteu assay. Data of each plant sample and averages of all species (A-E) are presented as mg of Gallic acid per gram of dry weight (gdw). Statistical analyses of all average data were performed using ANOVA. All average data resulted [file 6782472.f1.pdf]

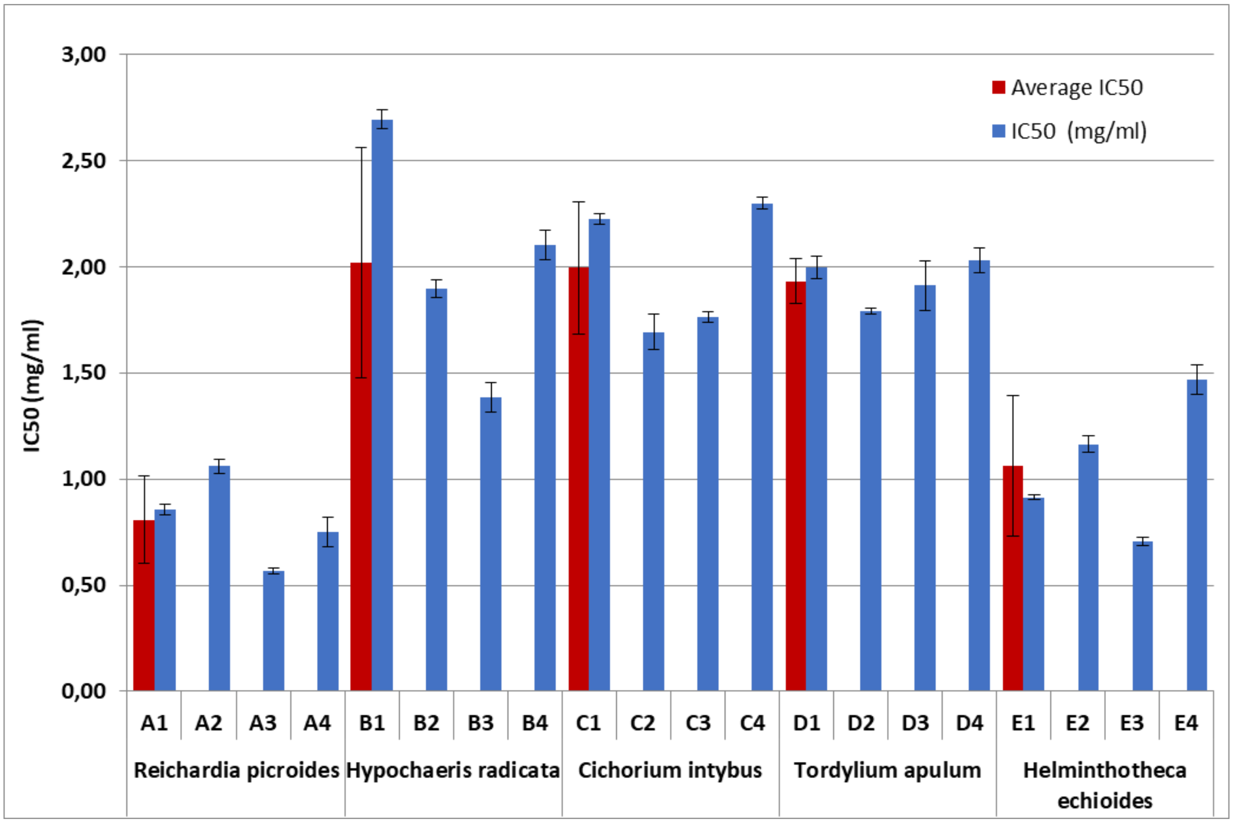

Figure S1. DPPH assay of the crude plant extracts. Data of each plant sample and averages of all species (**A-E**) are presented as IC_{50} i.e., the inhibition concentration that halves the DPPH radical activity. Lower IC_{50} values indicate higher antioxidant capacity. Statistical analyses of all average data were performed using ANOVA. Values for **A** and **E** resulted not statistically different from each other but different from all other values ($p < 0.01$) except **E** versus **B** ($p<0.05$).

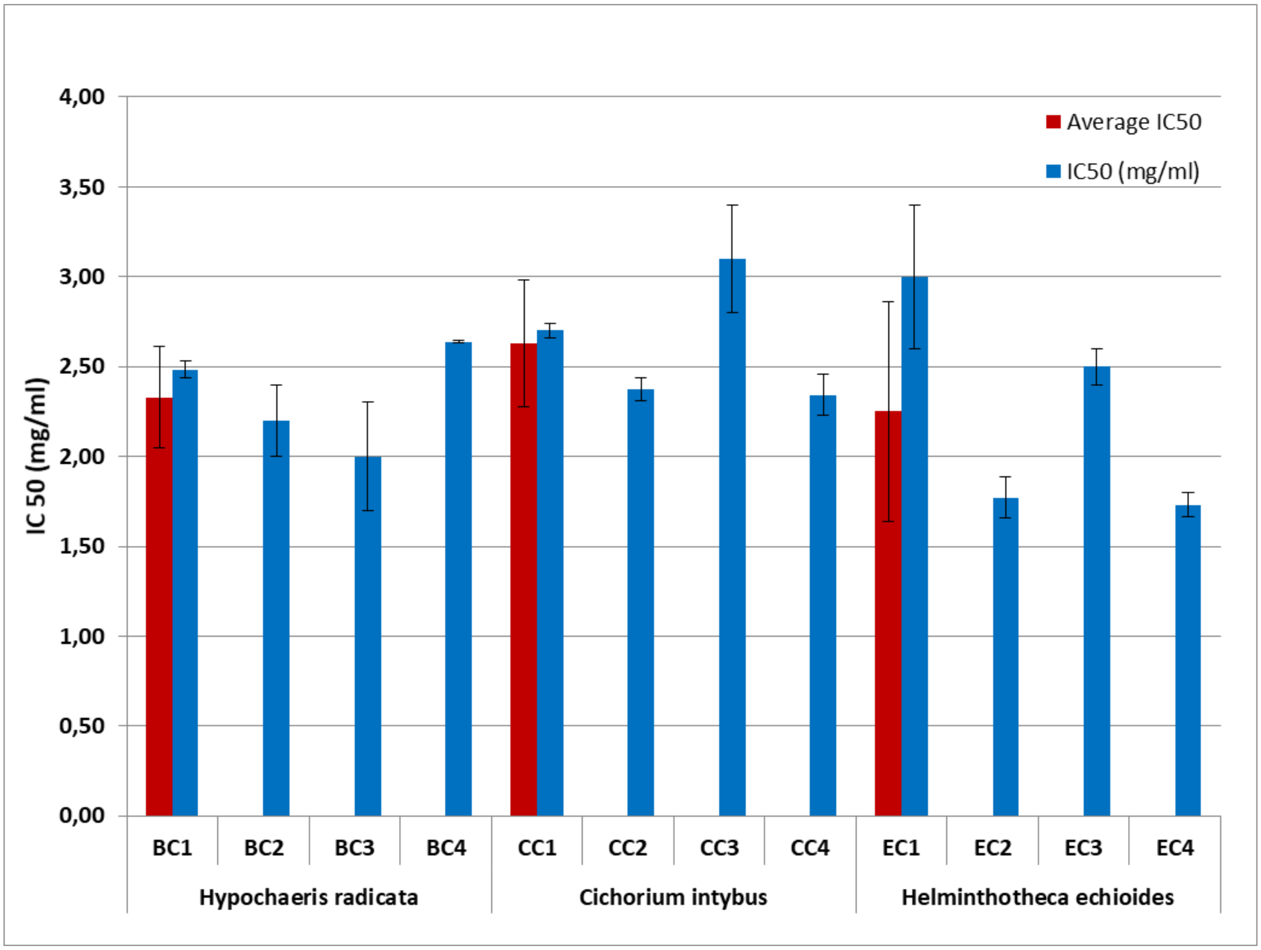

Figure S2. DPPH assay of the cooked plant extracts. Data of plant samples and averages of species (**BC**, **CC** and **EC**) are presented as IC_{50} i.e., the inhibition concentration that halves the DPPH radical activity. Lower IC_{50} values indicate higher antioxidant capacity. Statistical analyses of all average data were performed using ANOVA. Value resulted not statistically different from each other.

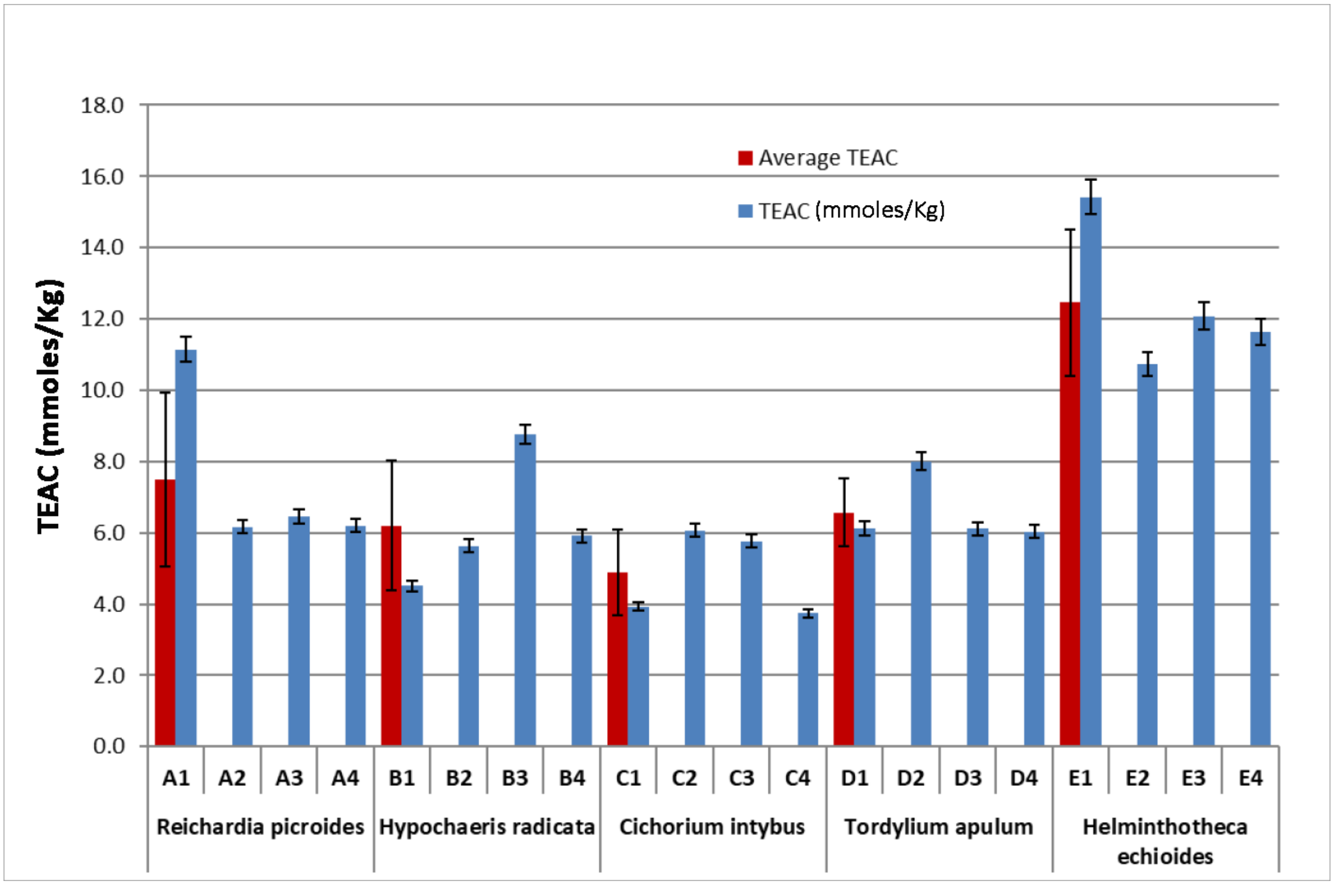

Figure S3. ABTS assay of the crude plant extracts. Data of each plant sample and averages of all species (**A-E**) are presented as TEAC i.e., mmoles of Trolox equivalent *per* Kilogram of fresh weight (Kgfw). Statistical analyses of all average data were performed using ANOVA. All average data resulted not statistically different from each other except for **E** that was statistically different from **A** ($p < 0.05$), **B** ($p < 0.01$), **C** ($p < 0.001$) and **D** ($p < 0.01$).

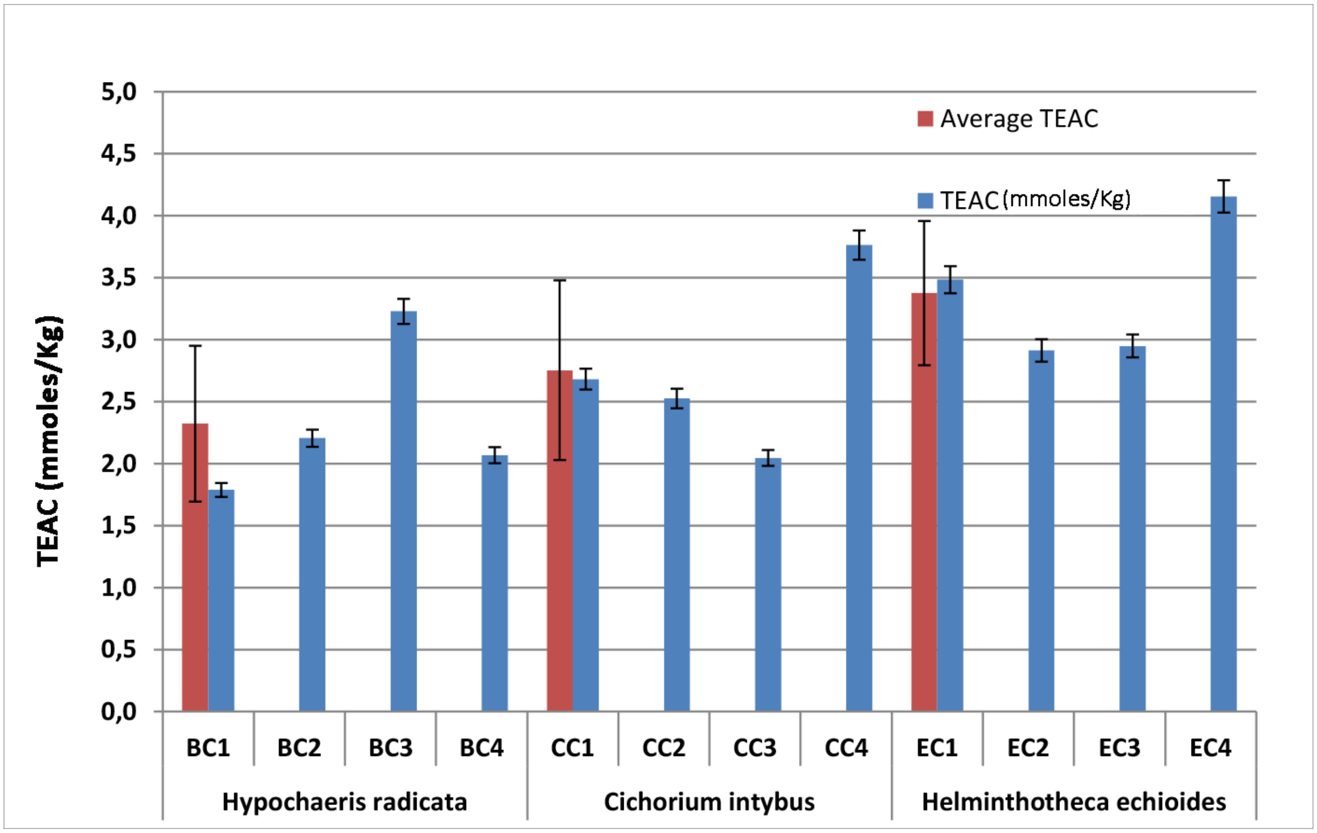

Figure S4. ABTS assay of the cooked plant extracts. Data of plant samples and averages of species (**BC**, **CC** and **EC**) are presented as TEAC i.e., mmoles of Trolox equivalent *per* Kilogram of fresh weight (Kgfw). Statistical analyses of all average data were performed using ANOVA. All average data resulted not statistically different from each other.

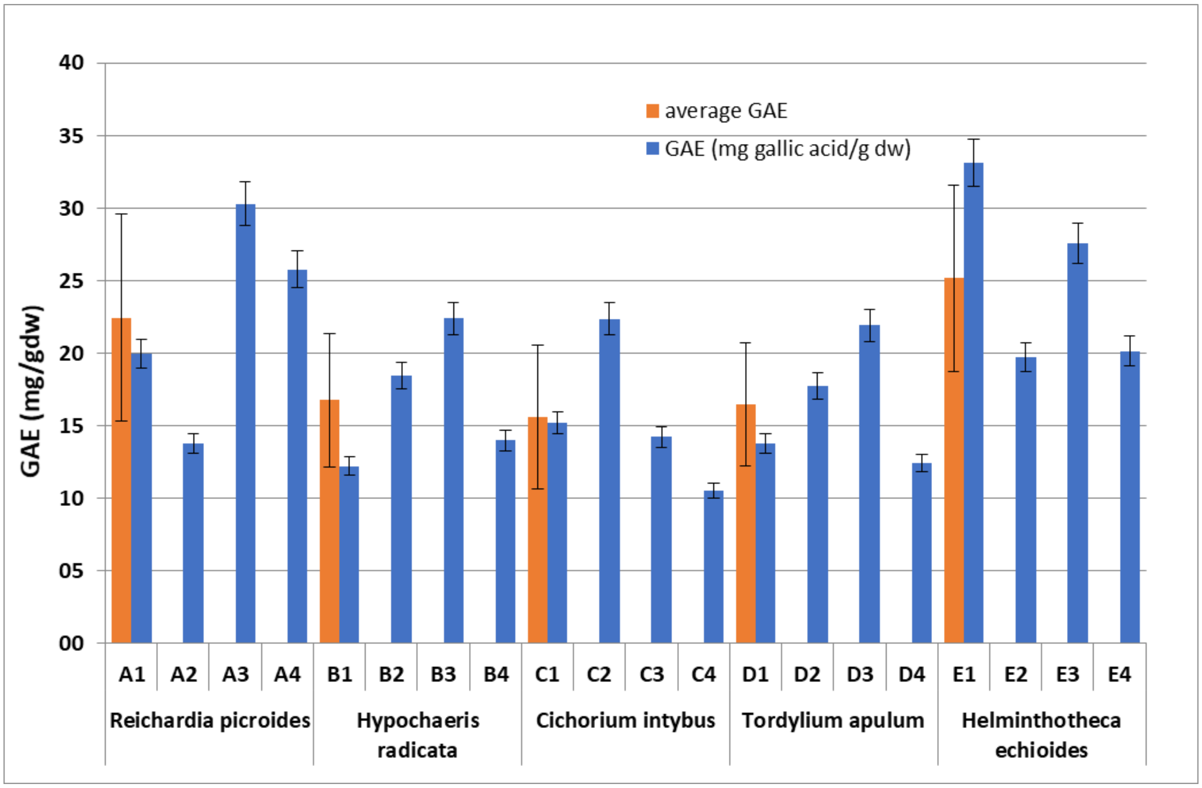

Figure S5. Total Phenolic Content of the crude plant extracts determined using the Folin-Ciocalteu assay. Data of each plant sample and averages of all species (**A-E**) are presented as mg of Gallic acid *per* gram of dry weight (gdw). Statistical analyses of all average data were performed using ANOVA. All average data resulted not statistically different from each other except to some marginal differences between **E** and **B** or **C** and **D**.

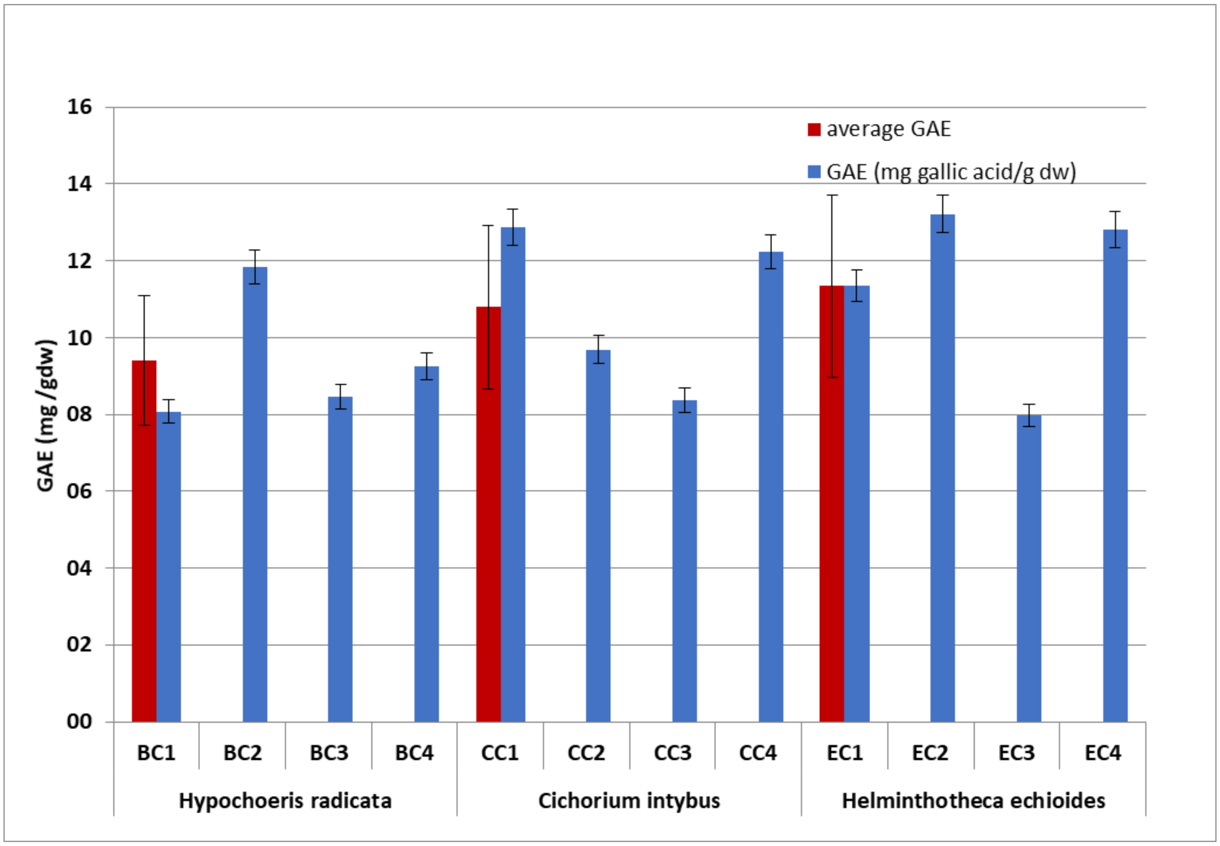

Figure S6. Total Phenolic Content of the cooked plant extracts determined using the Folin-Ciocalteu assay. Data of plant samples and averages of species (**BC**, **CC** and **EC**) are presented as mg of Gallic acid *per* gram of dry weight (gdw). Statistical analyses of all average data were performed using ANOVA. All average data resulted not statistically different from each other.
